# Trainable WEKA (Waikato Environment for Knowledge Analysis) Segmentation Tool: Machine-Learning-Enabled Segmentation on Features of Panoramic Radiographs

**DOI:** 10.7759/cureus.21777

**Published:** 2022-01-31

**Authors:** Nitin Kanuri, Ahmed Z Abdelkarim, Sonali A Rathore

**Affiliations:** 1 Oral Diagnostic Sciences, Mills E. Godwin High School, Richmond, USA; 2 Oral and Maxillofacial Radiology, University of Texas Health Science Center at San Antonio, San Antonio, USA; 3 Oral Diagnostic Sciences, Virginia Commonwealth University, Richmond, USA

**Keywords:** panoramic radiographs, dice score, jacard index, gaussian, segmentation

## Abstract

Introduction: Segmentation of dental radiographs is a comprehensive subject in oral care and diagnosis. It is the process of delineating anatomical structures to simplify the diagnostic process for oral and maxillofacial radiologists.

Purpose: This paper will provide an in-depth analysis of the latest benchmarks in oral imaging by studying the segmentation of panoramic radiographs using Trainable WEKA (Waikato Environment for Knowledge Analysis) Segmentation (TWS). The aim of this research is to accurately automate segmentation where it can be implemented on a large scale of clients in order to simplify radiological diagnosis.

Methods and Materials: The experimentation was conducted by modifying open-source radiographs from UFBA UESC DENTAL IMAGES dataset. In order to simulate realistic conditions such as noise affecting regions of interest, panoramic radiographs were degraded and blurred with Gaussian noise. Accuracy was quantified by measuring the difference between the automated image and the dentist-annotated image using MorphoLibJ. To ensure the precision in results, automated predicted segmentations were observed by an oral maxillofacial radiologist and compared with the dentist-renditioning annotations of the panoramic radiographs (orthopantomograms).

Results: The TWS classifier on radiographs with an average of 32 teeth and greater (Dice value of 0.66) and an average of less than 32 teeth (F1 score of 0.59) was significant. The calculated t-value for the Jaccard index is 2.78 and the t-value for the Dice score is 2.81. The results, considering the statistical scores, were due to the independent variable. The radiographs with 32 teeth and greater had higher Intersection over Union scores and F1 scores because of less discrepancy in tooth alignment.

Conclusions: Segmentation of dental radiographs can be conducted by machine learning instead of manual segmentation.

## Introduction

Diagnostic sciences have many practical applications in dentistry allowing for dental professionals to screen patients for problems such as infection, bone loss, abscesses, tumors, and cysts. Simply put, without the use of radiological technologies, dental ailments cannot be detected until an increase of severity putting the patient at risk. There has been much research on automatically segmenting images; however, a successful algorithm is difficult to create due to variations of each dental radiograph. Another practical application of radiology in dentistry is the contribution to the accuracy of treatment plans. In other words, solely depending on specialist examination [1] can introduce bias into treatment. Thus, an algorithm for automatic segmentation has long been sought. According to a study in 2004, the automatic radiograph segmentation will assist specialists in detecting damaged teeth and identifying appropriate placement of dental implants [2]. This paper aims to develop and test an automatic segmentation using self-identified classifiers, which aims to accurately segment images using the Trainable WEKA (Waikato Environment for Knowledge Analysis) Segmentation (TWS) of the Fiji, which is the Just ImageJ (FIJI) platform. Panoramic radiographs, often called orthopantomograms, are panoramic scans of the upper and lower jaw, which provide a two-dimensional (2D) radiograph of the teeth and mandible. Panoramic radiographs are taken for several reasons, a few including assessment of the temporomandibular joint, evaluation of orthodontic treatment, and the regular oral health check-ups. The classifiers are trained using orthopantomograms from the UFBA UESC DENTAL IMAGES dataset.

Explanation of dental segmentation

Image segmentation is the partitioning of an image sample into several regions to simplify the image representation and its analysis [3]. Segmentation of dental radiographs is the process of delineating anatomical structures to simplify the diagnostic process for oral and maxillofacial radiologists. The segmented image plays a major role in the early detection of abnormalities to help specialists produce an accurate diagnosis [4]. This paper aims to develop an automatic machine learning-enabled method for segmentation of digital dental panoramic radiographs. The independent variable is the two categories that were made on the average number of teeth and the dependent variable is the Jaccard index and the Dice score.

Literature review

There have many been novel approaches to segmentation of images. Lira et al. (2014) [5] proposed a segmentation approach based off a supervised learning technique for texture recognition. It was proposed that the obtained data from feature extraction is inputted to a Bayesian classifier that has the ability to distinguish two classes of pixels. In another study, Kakehbaraei et al. (2018) proposed a five-part segmentation process consisting of obtaining cone beam computed tomography samples, enhancing the image, marker-controlled watershed-enabled segmenting of the teeth, applying a global threshold for the enamel segmentation, and using marker-controlled watershed for segmentation of the pulp [6]. In another study, Kumar et al. (2011) [7] propose a technique that avoids limitations to segment a three-dimensional dental model by first separating the gums and then separating the individual teeth of the model. Another novel approach to segmentation was proposed by Al-Sherif et al. (2012), which made use of a two-step thresholding segmentation technique and adapted off of a seam carving technique [8].

## Materials and methods

Construction of dataset

Details of Dataset

The images from the dataset were taken from the radiograph Sirona Orthophos XG 5 at the Diagnostic Center of the Southwest State University of Bahia [3]. All orthopantomograms were numbered and anonymized. Images were taken with a matrix size of 2440 × 1292 pixels taken in gray level.

Variation of Dataset

This dataset also consists of a variety of radiographs. Added features on panoramic radiographs complicate the process of segmentation because there is no homogeneity from a radiograph to another. Such differences include missing and broken teeth, crowns, and restorations to cite a few. The main challenges are varying levels of noise, image of the vertebral column, and low contrast [3]. Such problems require careful expert analysis; however, the classifier is trained using radiographs of all discrepancies and eliminates the concern of non-homogeneity. The data were split into two categories: a group with 32 or greater teeth, and another with less than 32 teeth. The independent variables are these two categories. The discrepancies between radiographs are shown in Table 1.

**Table 1:** Discrepancy of images in the dataset and numerical count of images [3].

Category	Discrepancy	Sample count	Average teeth
Average of 32 teeth or more	All teeth, restoration, dental appliance	73	32
	All teeth, restoration	220	32
	All teeth, dental appliance	45	32
	All teeth	140	32
	More than 32 teeth	170	37
Average of less than 32 teeth	Dental implant	120	18
	Missing teeth, restoration, dental appliance	115	27
	Missing teeth, restoration	457	29
	Missing teeth, dental appliance	45	28
	Missing teeth	115	28

Gaussian blur

**Figure 1:**
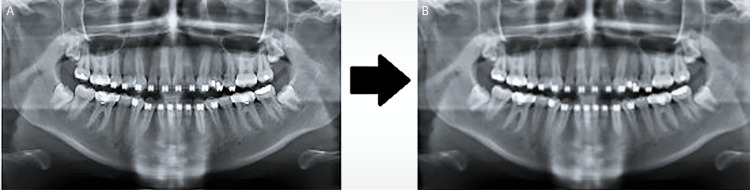
Example of filtering images using ImageJ's Gaussian filter. A: Original, B: image with Gaussian filter.

Image noise is a common problem in image processing in the medical imaging field. This is evident by the amount of literature aiming to reduce or circumvent it [9]. The ImageJ Gaussian blur filter was manually applied on each original image of dataset to give values closest to the gold standard. The Gaussian blur filter applies a convolution using a sigma (which is the radius of decay to exp(-0.5) 61 percent - the standard deviation sigma of the Gaussian) of a Gaussian function for dulling the image [10]. Figure 1 shows a visual of a radiograph being filtered using Gaussian blur.

Cropping images

**Figure 2:**
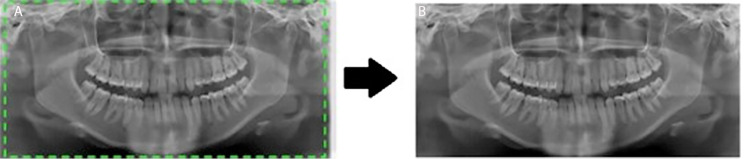
Depicting the process of cropping images to the region of importance. A: Original image (2440 × 1292 pixels), B: cropped image (1991 × 1127 pixels).

The original dimensions of the pictures were 2440 × 1292 pixels and they were resized to 1991 × 1127 pixels. The region of interest was determined by multiplying values of the pixel array elements by its corresponding binary matrix (from the oral annotation), resulting in the final 1991 × 1127 pixels image as shown in Figure 2.

Data were processed on machine with 16 GB RAM and 10 Intel Core i5 at 1.60 GHz (4.20 GHz at boost). The graphic card parameters are not necessary in the processing of this environment. Of the image dataset, a traditional 80-20 training/test split was used. The classifier in the TWS was trained to use characteristics of the first 1300 images and then tested on the final 200 images to produce accuracy metrics. 

Normalization

Normalization modifies the value of pixel intensity. Before images were processed, all images were subjected to the Enhance Contrast feature on ImageJ. The saturated pixel value was set to 0.3% and the settings for Normalize and Equalize Histogram were applied. Saturated pixels indicate the number of pixels in the image that are saturated. The Normalize option changes the pixel range of the image to the maximum range of 8-bit images such as these to 0-255 gray values. The Equalize Histogram option augments the sample with a histogram equalization making use of an algorithm that is programmed to calculate the square root of the histogram values.

ImageJ filters

All images were subjected to ImageJ’s TWS plugin [10]. The training implemented the use of the Random Forest algorithm in WEKA. Adapted from a previous study, the following filters were applied in the environment: Anisotropic diffusion, Lipschitz, Gabor, Hessian, Gaussian blur, Entropy, Maximum, Sobel filter, Difference of Gaussian filters, Median filter (minimum sigma = 1.0, maximum sigma = 4.0), and Membrane projections (membrane thickness 1 mm, membrane patch size = 10) [11]. These filters were applied to simulate the gold standard segmentation by a specialist [11].

MorphoLibJ (ImageJ plugin)

The classifier was applied to the testing dataset (200 images) and the final result was generated. The accuracy metrics were achieved using MorphoLibJ plugin for ImageJ. MorphoLibJ [12] is a collection of mathematical morphology methods created at INRA-IJPB Modeling and Digital Imaging lab. Given a source image S and a target image T, MorphoLibJ calculates the overlap agreement between two label images adapted after Serra and Vincent’s work [13]. The source image S was the dentist annotation of the radiograph compared with the target image T to obtain the results using the MorphoLibJ plugin.

Evaluation of the segmentation methods

Metrics for Performance Analysis

The Jaccard index is sometimes referred to as the Intersection over Union (IoU) or the Jaccard loss. The Jaccard index is a validation metric that is defined by the number of matching pixels over the total number of matching pixels and mismatching pixels [14]. The equation for this concept is J(A,B) = (|A B|)/(|A B|).

**Figure 3:**
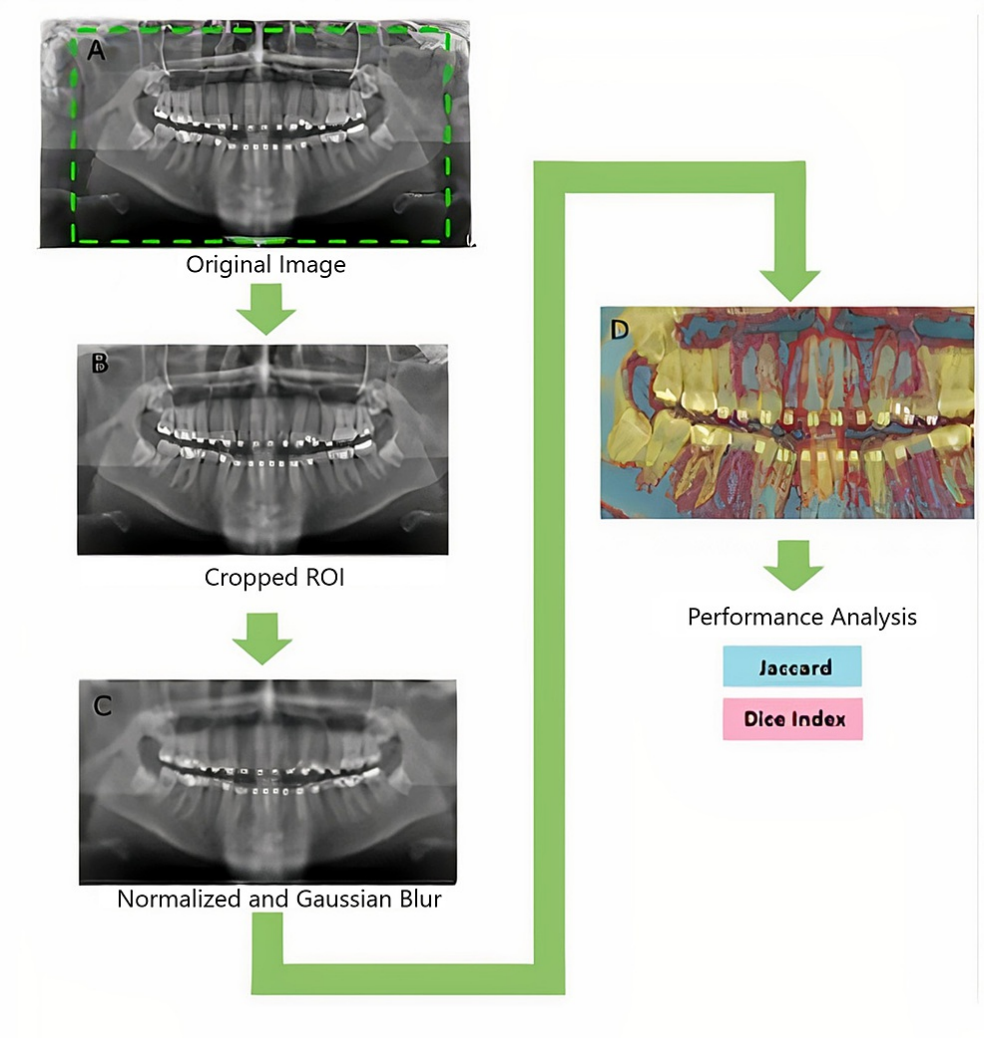
Flowchart of the process. A, B, C, and D show the sequence of the steps. ROI, region of importance.

The Dice similarity coefficient (DSC) is more commonly known as the Dice coefficient, but also referred to as the Sørensen-Dice index. The Dice coefficient is a statistical validation metric used for gauging the performance of manual segmentations and the spatial overlap accuracy between two samples. The equation for this is Dice(A,B) = (2|A B|)/(|A|+|Y|), where A and B are two sets [15]. When the set is surrounded by two vertical bars “|” it indicates cardinality of the set (the number of elements the set contains). This refers to the intersection of two sets [16]. The DSC is from a range of 0 to 1; 0 indicates no spatial overlap, whereas 1 indicates a complete overlap between two samples. The DSC is the same as the F1 score and this value is monotonic to the Jaccard index. A summary of the process is demonstrated in this chart (Figure 3).

## Results

The accuracy of the classifiers on either categories (images with 32 teeth or greater versus images with less than 32 teeth) using the IoU and F1 score metrics was analyzed using a t-test (0.05 level of significance and 198 degrees of freedom) and the results of the statistical analysis are shown in Table 2 and Table 3.

**Table 2:** Statistical analysis for the effect of categorical discrepancy of radiographs on Jaccard index (intersection over union) given the source image S and the target image T.

Descriptive info	Category of radiographs	
	Average of 32 teeth or more	Average of less than 32 teeth
Mean	0.524	0.425
Range	0.775	0.694
Minimum	0.039	0.025
Maximum	0.814	0.843
Variance	0.036	0.030
Standard deviation (SD)	0.189	0.173
1 SD	0.335-0.713	0.279-0.625
2 SD	0.146-0.901	0.105-0.799
3 SD	0-1.090	0-0.970
Number	100	100
Results for t-test	Average of 32 teeth or more vs average of less than 32 teeth. t = 2.78; p < 0.05	

**Table 3:** Statistical analysis for the effect of categorical discrepancy of radiographs on the Sorensen-Dice index (F1 score) given the source image S and the target image T.

Descriptive info	Category of radiographs	
	Average of 32 teeth or more	Average of less than 32 teeth
Mean	0.663	0.590
Range	0.899	0.664
Minimum	0.075	0.048
Maximum	0.975	0.915
Variance	0.034	0.032
Standard deviation (SD)	0.184	0.108
1 SD	0.478-0.847	0.410-0.771
2 SD	0.293-1.103	0.230-0.951
3 SD	0.109-1.217	0.050-1.131
Number	100	100
Results for t-test	Average of 32 teeth or more vs average of less than 32 teeth. t = 2.81; p < 0.05	

**Figure 4:**
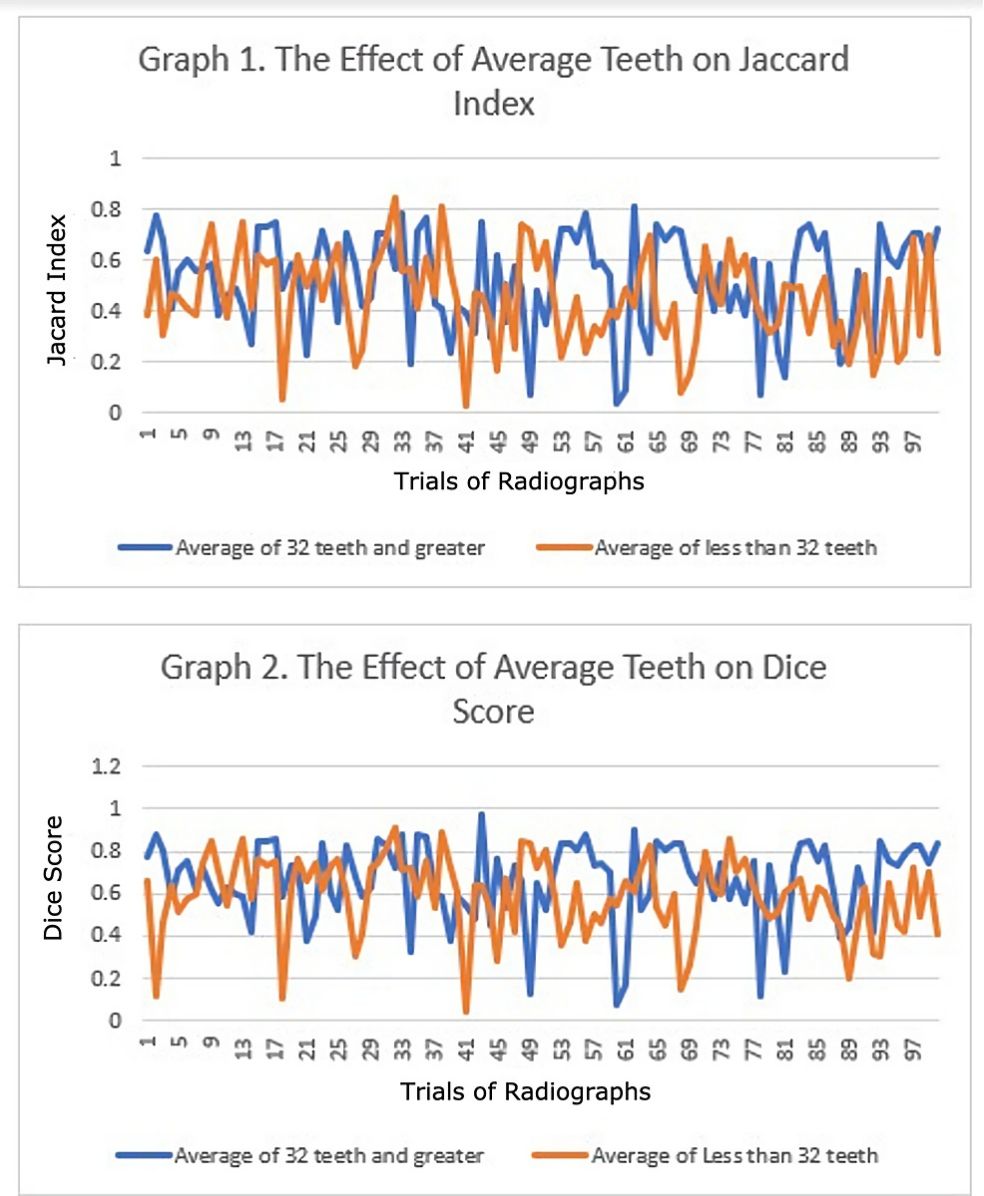
Graph 1 for Jaccard index and graph 2 for Dice score

It was hypothesized that the group images with 32 or more teeth would have the higher accuracy metrics (IoU and F1 score) because it is assumed that it is simpler for the classifier to train a radiograph with all of its teeth over a radiograph with missing teeth (found in many of the samples). The null hypothesis was that there is no statistically significant difference in the accuracy metrics of images with 32 teeth or greater versus images with less than 32 teeth. Since the calculated t-value is greater than the critical value, the null hypothesis is rejected, and the results are statistically significant. As seen in Table 2 and Table 3, the data are statistically significant as a result of the influence of the independent variable. Taking a closer look at the data, it can be concluded that the data were precise because the standard deviations shown in Table 2 and Table 3 had low values. The results of the Jaccard index and Dice score, however, changed from image to another as seen in Graphs 1 and 2 (Figure 4).

## Discussion

The purpose of this experiment is to automatically segment panoramic radiographs using TWS to simplify the work of dental specialist in deciding treatment plans. It is especially useful in telemedicine in pandemic times. Machine learning tools such as the TWS can be trained to automatically segment images with formidable accuracy metrics as shown in Table 2 and Table 3. From the machine-segmented radiographs obtained from this program, dental specialists have the option to formulate a possible treatment plan if necessary [3]. Many difficulties arise when trying to accurately train the machine to segment these images such as radiograph non-homogeneity and other obstacles such as degradation of image quality and random Gaussian blur that varies from radiograph to another. This is seen from comparing an image set to another in Graphs 1 and 2. However, the data were precise and the results were significant. Many more problems also are a result of an unsuccessful automated segmentation such as limited automated resources and programs. Using the diverse 1,500 orthopantomogram dataset, classifiers were trained to separate between tooth and background on the TWS platform. Data were reported using MorphoLibJ plugin that calculated Jaccard index (IoU) and Dice score (F1 score). The results were calculated for 100 images from the first category of images with an average of 32 teeth and greater and for 100 radiographs from the second category of radiographs with less than an average of 32 teeth.

Sources of error

There were few sources of error in this experiment which was meant to procure exact results. One major one was a possible chance of researcher bias when selecting the regions to teach the classifier of the difference between tooth and background. To improve this experiment, a larger number of trials would suffice in order to receive more exact data. Accuracy and consistency of the experiment was monitored by two oral and maxillofacial radiologists.

Future research

Much future research is needed when it comes to such complexity of oral and maxillofacial region, especially when it comes down to the segmentation of teeth. An identical experiment such as this can be repeated except that it could be done using one of the following types of dental radiographs such as bitewing, periapical, full mouth survey, and occlusal radiographs. There is also much research being done on the application of Convolutional Neural Networks (CNN) on the automated segmentation and number of teeth in dental radiographs. Such research is seen in Ref. [17], which is an evaluation of deep learning methods that talk about their contributions and other relevant information.

## Conclusions

The purpose of this study is to prove that machine learning can accurately be used to automatically segment dental radiographs. The results of this study prove that panoramic radiographs with 32 teeth generally will have a higher accuracy segmentation than radiographs with less than 32 teeth. This discrepancy in accuracy leads to a possibility of further research. Panoramic radiographs with 32 teeth have a greater homogeneity and a full set of teeth, making it easier for the machine to segment those types of radiographs. This will provide dentists with a powerful tool for image analysis and interpretation. Computer-aided teeth detection and numbering simplifies the process of filling out digital dental charts. Machine learning for segmentation of dental radiographs can be conducted automatically instead of manually. This will provide dentists with a powerful tool for image analysis and interpretation. Computer-aided teeth detection and numbering simplifies the process of filling out digital dental charts. Further development in the future will offer an important role in the development of automatic diagnosis platforms.
